# Scalable Self-Assembly of Composite Nanofibers into High-Energy-Density Li-Ion Battery Electrodes

**DOI:** 10.1021/acsnano.4c07602

**Published:** 2024-09-19

**Authors:** Heng Wang, Yuling Xiong, Kate Sanders, Sul Ki Park, Jeremy J. Baumberg, Michael F. L. De Volder

**Affiliations:** † Department of Engineering, University of Cambridge, Cambridge CB3 0FS, U.K.; ‡ Department of Physics, University of Cambridge, Cambridge CB3 0HE, U.K.

**Keywords:** Li-ion battery, self-assembly, alignment, nanofibers, vanadium pentoxide, roll-to-roll coating

## Abstract

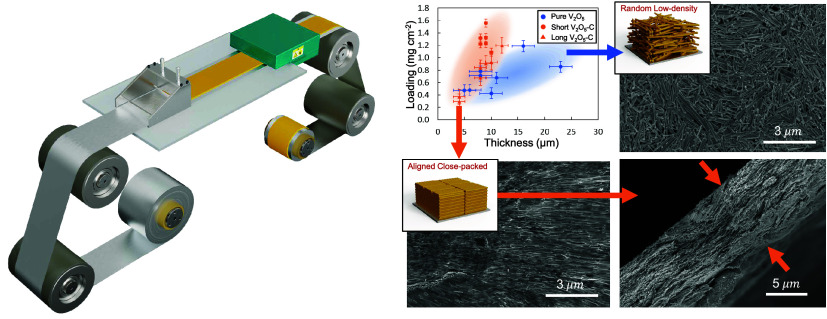

The application of nanosized active particles in Li-ion batteries has been the subject of intense investigation, yielding mixed results in terms of overall benefits. While nanoparticles have shown promise in improving rate performance and reducing issues related to cracking, they have also faced criticism due to side reactions, low packing density, and consequent subpar volumetric battery performance. Interesting processes such as self-assembly have been proposed to increase packing density, but these tend to be incompatible with scalable processes such as roll-to-roll coating, which are essential to manufacture electrodes at scale. Addressing these challenges, this research demonstrates the long-range self-assembly of carbon-decorated V_2_O_5_ nanofiber cathodes as a model system. These nanorods are closely packed into thick electrode films, exhibiting a high volumetric capacity of 205 mA h cm^–3^at 0.2 C. This surpasses the volumetric capacity of unaligned V_2_O_5_ nanofiber electrodes (82 mA h cm^–3^) under the same cycling conditions. We also demonstrate that these energy-dense electrodes retain an excellent capacity of up to 190.4 mA h cm^–3^(<2% loss) over 500 cycles without needing binders. Finally, we demonstrate that the proposed self-assembly process is compatible with roll-to-roll coating. This work contributes to the development of energy-dense coatings for next-generation battery electrodes with high volumetric energy density.

## Introduction

The increasing prevalence of Li-ion batteries (LIBs) in various applications, from consumer electronics to electric vehicles, demands continuous improvements in LIB energy and power density.^1−3^ In pursuit of higher volumetric energy density, researchers are focusing on two primary strategies: the development of LIB materials with inherently higher energy density and the more efficient utilization of existing materials. The latter approach involves increasing the areal loading^4−9^ or compressing materials into denser structures through calendering, a standard industrial practice. The materials used inherently limit the packing density. For instance, spherical battery particles with a uniform size distribution can achieve a maximum theoretical packing density of 74% (in FCC or HCP arrangements). Although higher packing densities are attainable using bimodal particle distributions, achieving these higher densities requires substantial calendering forces, which can lead to the cracking of the active materials.^10^ In comparison, hexagonal close-packed cylinders can achieve a theoretical packing density of 91%. The issue with this approach is that calendering is unable to change the orientation of rod-shaped battery particles, and therefore, other approaches need to be developed to work in conjunction with rod-shaped particles.

One promising approach to organizing rod-shaped materials is ‘self-assembly’, where materials spontaneously form ordered structures that correspond to a thermodynamic minimum under suitable conditions, often driven by weak and reversible interactions. Electrodes created through self-assembly have been successfully developed by using various techniques. Our group has previously shown that drop casting can be used to assemble TiO_2_–rGO composite nanowires into thick films for electrodes, enabling stable cycling.^11^ Additionally, other research teams have explored methods such as electrostatic-assisted self-assembly combined with freeze-drying,^12,13^ layer-by-layer stacking via vacuum filtration,^14^ and self-densification via organic layer encapsulation.^15^

However, the scalability of these methods, particularly their compatibility with commercial manufacturing techniques like roll-to-roll coating, remains a significant challenge, which hinders their practical large-scale application.^3,16^ Here, we focus on the design of scalable self-assembled LIB cathodes and demonstrate that (i) self-assembly can be used to pack cathode nanorods into aligned and dense structures; (ii) this process is compatible with the large-scale roll-to-roll coating and calendering; (iii) self-assembly allows for both improvements in volumetric energy density and cycling lifetime.

In this article, we use vanadium pentoxide (V_2_O_5_) as a cathode model system in combination with metal anode batteries. V_2_O_5_ is attractive for this application because it is relatively straightforward to synthesize into high aspect ratio nanorods. In addition, it has displayed high capacity^17−19^ and stable crystal structure under a variety of applications,^20−22^ and low cost. We optimize a template-free method to synthesize V_2_O_5_–carbon composite (V_2_O_5_–C) nanofibers, where the carbon additive is important to achieve good self-assembly behavior and, at the same time, improve electric conductivity.

In our work, we demonstrated that our self-assembled V_2_O_5_–C electrodes can achieve a high volumetric capacity (205 mA h ) and a capacity retention of 98.1% after 500 cycles in the absence of binders and conductive additives, significantly outperforming unaligned pure V_2_O_5_ electrodes (82.0 mA h cm^–3^). Using a continuous roll-to-roll coating process, we demonstrated the manufacturing of 4 m of self-aligned electrode, and we found that calendaring can be combined with self-assembly to create higher energy density electrodes. This self-assembly approach provides insights into how nanomaterials can be structured into LIB electrodes without compromising the volumetric energy density or manufacturing scalability.

## Results and Discussion

### Material Synthesis and Characterization

**Figure 1:**
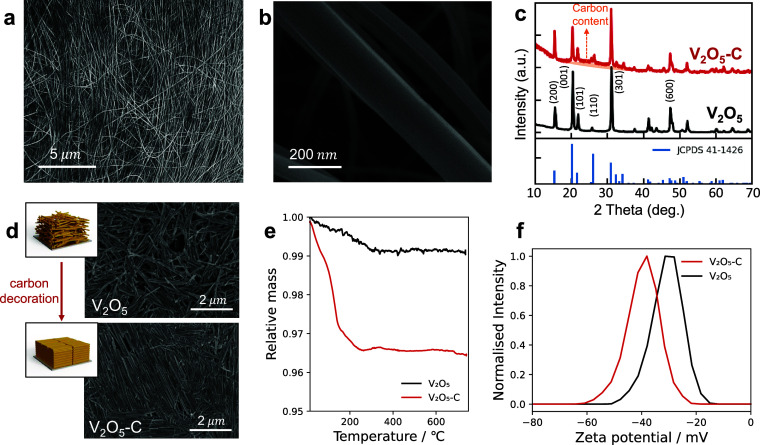
Characterizations of V_2_O_5_–C NFs: (a) SEM images of synthesized V_2_O_5_–C NFs at low magnification and (b) at high magnification. (c) XRD patterns of the V_2_O_5_–C NFs compared to pure V_2_O_5_ NFs. (d) Schematic and SEM top view of nonaligned, pure V_2_O_5_ NFs (top) versus aligned, self-assembled V_2_O_5_–C NFs (bottom). Both samples were prepared by drop-casting suspensions of NFs in water at a concentration of 5 mg/mL. (e) Representative TGA of the V_2_O_5_–C NFs sample and the pure V_2_O_5_ NFs sample in air. (f) Zeta potential of the NF samples in DI water (mean zeta potential is −30.7 ± 5.6 mV for V_2_O_5_, and −39.5 ± 5.8 mV for V_2_O_5_–C).

The V_2_O_5_–C nanorods are synthesized by adding 5 wt % of graphene oxide (GO) into V_2_O_5_ sol (mixture of 1.2 g V_2_O_5_ powder, 86 mL of DI water, and 17 mL 30 wt % H_2_O_2_) and undergo a 96 h hydrothermal treatment (see Experimental Section). SEM images show the resulting composite nanofibers (NFs) have diameters of 100–200 nm and lengths exceeding 20 μm (Figure 1a,b). The crystal and chemical structure of the V_2_O_5_–C NFs have been characterized using XRD. Their XRD pattern (Figure 1c) matches with an orthorhombic V_2_O_5_ crystal phase (JCPDS no. 41-1426), albeit with a notable attenuation in the intensity of the (110) plane and enhancement in the intensity of the (301) plane due to the preferential crystal growth along [110] direction.^23,24^ In contrast to pure V_2_O_5_ NFs, a relatively weak and broad shoulder is observed in our V_2_O_5_–C NFs at 2θ ∼ 25° which corresponds to the carbon content within the composite. The self-assembly behavior of V_2_O_5_–C NFs is conducted using a drop-casting method on silicon chips, following a previously published procedure.^20^ Initially, dried V_2_O_5_–C NFs powders are mixed with water to achieve a 5 mg/mL concentration and then sonicated to ensure thorough dispersion. Subsequently, 100 μL of this suspension is deposited onto a 1 cm × 1 cm silicon chip and dried at 60 °C on a hot plate. SEM analysis (Figure 1d) of the resultant V_2_O_5_–C film reveals an aligned structure, in stark contrast to the random, nonaligned structure observed in the film prepared under identical conditions with pure V_2_O_5_ NFs. This confirms that the addition of carbon significantly enhances the self-assembly process. TGA performed in the air (Figure 1e and Note S1) shows that V_2_O_5_–C NF samples synthesized with GO have a greater mass decrease below 300 °C compared to pure V_2_O_5_ NF samples. However, the overall mass decrease in the V_2_O_5_–C nanowire sample is still quite small 97 ± 1%, suggesting that the remaining carbon species in the nanofiber structure likely contributed to about 2 wt %. In addition, the V_2_O_5_–C NF sample synthesized with GO has a more negative zeta potential of −39.5 ± 5.8 mV in DI water compared to that of pure V_2_O_5_ NF, indicating improved colloidal stability (Figure 1f). This enhanced negative surface charge is likely due to oxidized carbon functional groups (carboxylic/hydroxyl), which prevent quick and random agglomeration of nanofibers, allowing time and freedom for self-alignment.^25−27^

### Self-Assembly of V_2_O_5_–C NFs

Previous work on carbon-coated titanium oxide nanorods suggests that charges on oxidized carbon particles play a role in the self-assembly process.^11,27^ These surface charges, confirmed by the zeta potential (Figure 1f), can be modified by changing the pH of the solution around the p*K*_a_ of our V_2_O_5_–C NFs. Therefore, we started by studying the effects of pH on self-assembly behavior when drop-casted on silicon substrates (Figure S3). Initially, we prepared suspensions of V_2_O_5_–C NFs at a concentration of 5 mg/mL in water (adjusted to pH 4, pH 7, and pH 10) and adjusted the pH levels by HCl or NaOH. The morphology of the resulting drop-casted samples is examined under SEM and the alignment is quantified by alignment ratio η, which is calculated by taking a Fast Fourier transform (FFT) of SEM images of nanorod films^28,29^ to extract periodic patterns and their alignment (calculation method detailed in Note S2 and Figure S2).^27,30^ The absolute value of the alignment ratio does not reflect a physical quantity and is used only for comparison between samples. Therefore, when comparing images, η is normalized to 1 for the most aligned, and 0 for the least aligned sample. For the aqueous NF slurry (Figure S3), V_2_O_5_–C films show much better alignment (packing) than pure V_2_O_5_ films, and the alignment of the V_2_O_5_–C NF film is maintained across a broad range of pH from 4 to 10 with η maximized at pH 7 (Figure S3c), similar to previous work.^20^ Next, we tested the effect of using nonaqueous dispersions (Figure S4) using ethanol and NMP, however, both V_2_O_5_–C NF and pure V_2_O_5_ NF film made using these solvents show random alignment.

**Figure 2:**
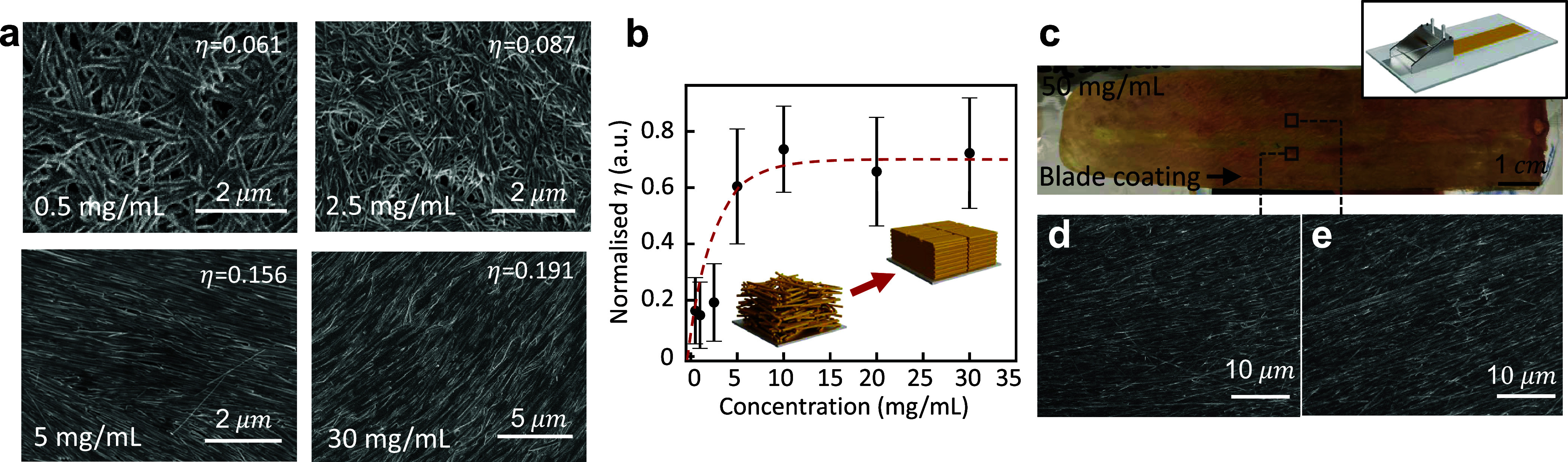
Self-assembly behavior of V_2_O_5_–C NFs in an aqueous environment. (a) SEM images of drop-casted V_2_O_5_–C NFs films prepared at four different concentrations (0.5, 2.5, 5, and 30 mg/mL) alongside the calculated alignment ratio η. (b) Normalized alignment ratio as a function of concentration, with a red dashed line provided for guidance. Error bars represent the standard deviation calculated from the alignment ratios of three different positions on each sample. (c) Bench-top blade coating of 50 mg/mL V_2_O_5_–C NFs on an Al substrate. Inset: schematic of the benchtop blade coating setup. (d, e) SEM images taken from two different positions of sample in (c), showing alignments of NFs parallel to the coating directions.

Finally, we explore the effect of the V_2_O_5_–C NFs concentrations on alignment in water suspensions (0.5, 1, 2.5, 5, 10, 20, and 30 mg/mL), comparing against reference V_2_O_5_ NF samples at 5 and 50 mg/mL. Figure 2 shows the relationship between the concentration of the V_2_O_5_–C NFs suspension and the alignment ratio η determined by the FFT method. V_2_O_5_–C NFs show random orientation after drop-casting when the dispersion is dilute, and the alignment improves with the concentration increase. The NFs start to show self-assembly behavior at 2.5 mg/mL and high alignment is obtained at concentrations above 5 mg/mL (Figures 2a and S5), suggesting a critical concentration for achieving aligned nanofibers between these two concentrations. At concentrations of 30 mg/mL, V_2_O_5_–C NFs show excellent alignment uniformity over larger areas. This concentration dependency is consistent with Onsager’s theory,^31^ which characterizes phase diagrams depending on the concentration and aspect ratio of colloid nanoparticles, essentially predicting that high aspect ratios and concentrations favor alignment. In the case of pure V_2_O_5_ NF without carbon coating, drop-casted film alignment only becomes discernible at concentrations of 50 mg/mL (Figure S6). This significantly higher critical concentration for V_2_O_5_ NFs to show self-assembly behavior (i.e., isotropic-to-nematic phase transition) in comparison to V_2_O_5_–C again suggests that the surface chemistry of the carbon coating facilitates alignment.

Self-assembly is often studied on small centimeter square areas, whereas battery applications require large square meters of scalable manufacturing. Therefore, we investigate the interaction between the manufacturing and self-assembly processes with the aim of creating scalable, high-packing-density coatings. We first use a linear knife-over coater with a 50 mg/mL V_2_O_5_–C aqueous suspension on an aluminum foil current collector (see Experimental Section). Figure 2c shows a 14 × 4 cm blade-coated V_2_O_5_–C NF electrode. SEM images confirm that the self-assembly behavior of V_2_O_5_–C NFs is preserved during the blade coating process, resulting in well-aligned nanofibers (Figure 2d,e). Interestingly, the nanofibers show a clear preference for alignment parallel to the coating direction throughout the electrode. This suggests that shear forces applied by the coating head affect V_2_O_5_–C NFs alignment orientation and that this alignment does not relax during the drying process. By comparison, blade-coated pure V_2_O_5_ nanofibers did not show any alignment (Figure S7), even when using high suspension densities of 50 mg/mL.

**Figure 3:**
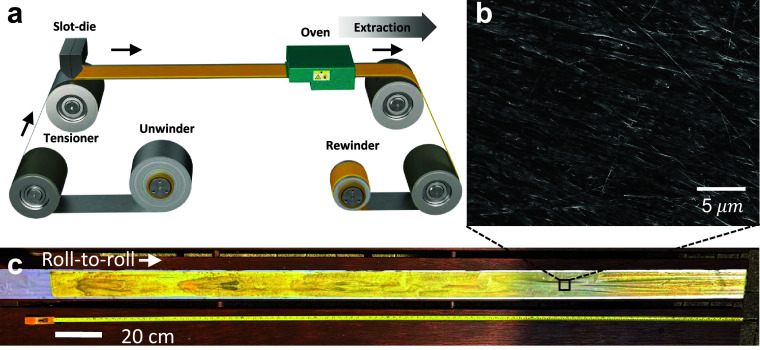
Roll-to-roll coating of V_2_O_5_–C NFs. (a) Schematic of the roll-to-roll slot die coating process using 25 mg/mL V_2_O_5_–C NFs aqueous dispersion. (b) SEM images showing highly aligned V_2_O_5_–C films achieved in our roll-to-roll coater (c) Photo of a continuously coated aligned V_2_O_5_–C NFs electrode on an aluminum substrate.

Encouraged by these initial results, we moved our process to a prepilot continuous roll-to-roll slot-die-coating process. This process is used for manufacturing battery electrodes in the industry and is, therefore, an important benchmark for these experiments. For this study, 80 mL of 25 mg/mL V_2_O_5_–C aqueous suspension is prepared and then slot-die coated continuously on a roll-to-roll tool (Figures 3a and S8) at a line speed of 0.1 m/min and dried using a combination of convection ovens and IR-heaters. A total length of 4 m and 11 cm wide V_2_O_5_–C NFs cathode film was produced with a stable continuous coating process, as shown in Figure 3c (the coating length was limited by the amount of suspension that we were able to produce). SEM images (Figures 3b and S9) taken at three different regions on the electrode confirm that a close packing of NFs is achieved by self-assembly on a several-meter-long electrode. The orientation of the aligned NFs shows a slightly weaker correlation with the coating direction, likely due to different amounts of shear force induced by the slot-die coating head compared to that of the knife-over coater. For battery electrodes, the packing density rather than orientation with the coating direction is important. For other applications where a uniform orientation is desired over large areas, it is possible that increasing the speed of the slot-die coating process and, therefore, the shear forces would further improve the uniformity in orientation. However, our coating speed was limited by the drying speed in our ovens.

**Figure 4:**
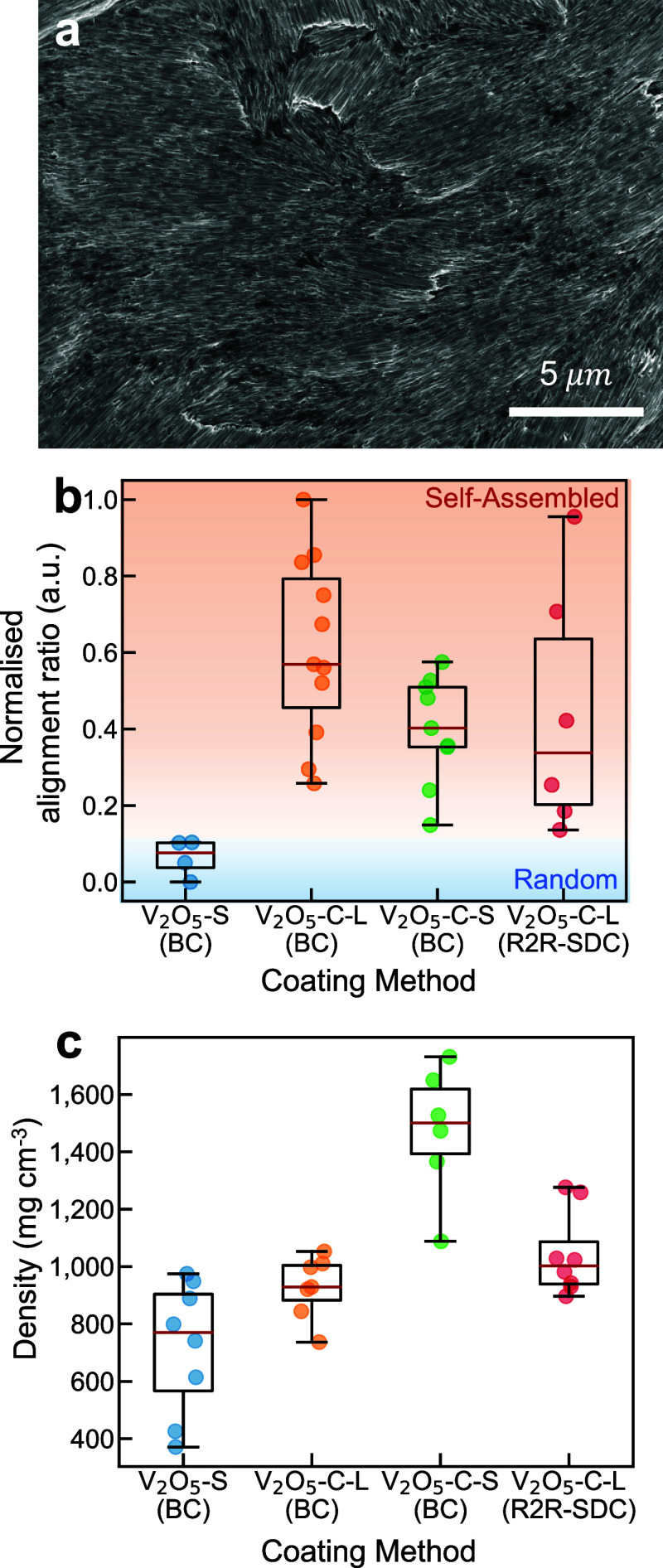
Effect of aspect ratios on nanofiber self-assembly. (a) SEM image of a short V_2_O_5_–C nanofiber film prepared using blade coating (ground before coating). (b) Normalized alignment ratio comparison between different coating methods. BC: (benchtop) blade coating; R2R-SDC: R2R slot-die coating. V_2_O_5_-S: short V_2_O_5_ nanofibers; V_2_O_5_–C-L: long V_2_O_5_ with carbon decoration; V_2_O_5_–C-S: short V_2_O_5_ with carbon decoration. (c) Density measured from different blade-coated samples.

Furthermore, Onsager’s theory predicts that the aspect ratio of the nanorods should influence their alignment, and therefore short V_2_O_5_–C NFs (V_2_O_5_–C-S) were prepared by an additional step of grinding to a shorter length before sonication into slurry suspension for blade-coating. As observed in Figure 4a, these shorter NFs form smaller domains within which the NFs align with each other. However, each domain exhibits different alignment orientations, indicating a weaker dependence of long-range alignment on the shear force applied by the coating blade. Examining the cross-section of films coated with both the long V_2_O_5_–C NFs (V_2_O_5_–C-L) and V_2_O_5_–C-S (Figure S10), we observed that the films with short NFs, while only displaying short-range order, are thinner and denser compared to those with long NFs when using the same NF dispersion concentration. This difference is likely because shorter NFs can more effectively orient and align whereas using fibers longer than the persistence length may introduce imperfections in the packing.

Finally, we compared the effects of different coating methods (blade coating vs slot die) and the NF lengths on the alignment. As shown in Figure 4b, the alignment behavior of V_2_O_5_–C can be maintained in both blade coating and slot-die coating. This contrasts with the random orientation of V_2_O_5_ NFs in the absence of carbon content. Notably, the alignment ratio is affected not only by the orientation but also by other factors such as detached surface NFs and multiple domains with varied alignments, leading to variability in alignment ratios (Figure S11). Further, the differences in packing density across each sample are validated by density measurements presented in Figure 4c, where a 2-fold increase of packing density is achieved through the self-assembly of short V_2_O_5_–C NFs compared to the random configuration of V_2_O_5_ electrodes. Although a general trend suggests that the electrodes with aligned nanofibers have higher packing densities than those with randomly oriented nanofibers, a direct correlation should not be assumed. The alignment ratios are derived from localized micron-scale regions under SEM, measuring only the lateral packing of nanofibers, while packing density measurements are across larger centimeter-scale areas accounting for three-dimensional packing.

### Electrochemical Characterization

**Figure 5:**
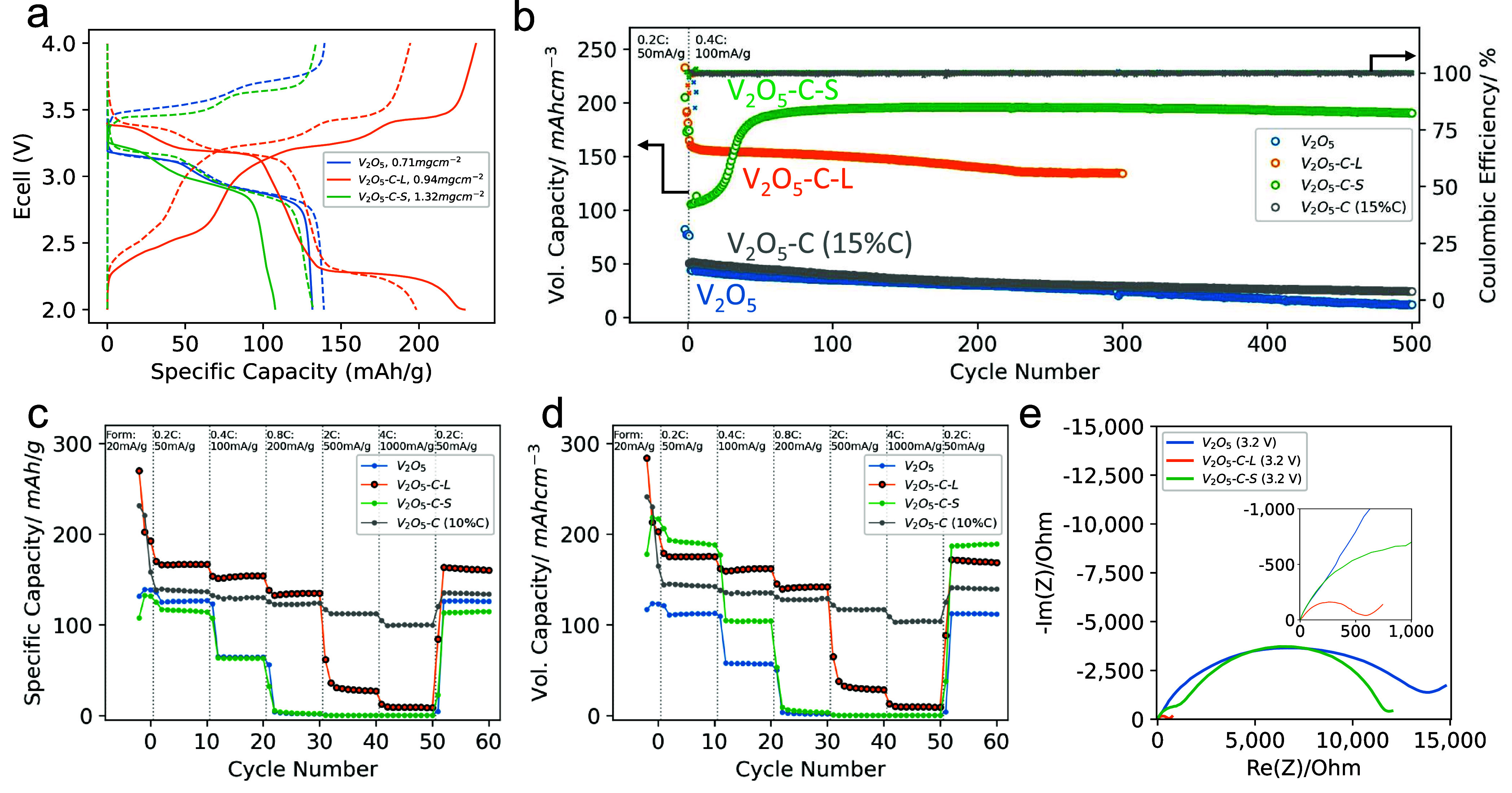
Electrochemical measurements. (a) Voltage curves for the first formation cycle (solid lines) and second formation cycle (dashed lines) at 20 mA g^–1^. (b) Volumetric capacity measurement of V_2_O_5_, V_2_O_5_–C-L, and V_2_O_5_–C-S, V_2_O_5_–C with 5% binder and 15% Super P over 500 cycles. (c) Gravimetric rate performance, and (d) volumetric rate performance of V_2_O_5_, V_2_O_5_–C-L, and V_2_O_5_–C-S, V_2_O_5_–C with 5% binder and 10% Super P conductive additive. (e) Electrochemical impedance spectroscopy after the formation.

Next, we investigate the battery performance of three types of electrodes: Pure V_2_O_5_ NFs (V_2_O_5_), long V_2_O_5_–C NFs (V_2_O_5_–C-L), and short V_2_O_5_–C NFs (V_2_O_5_–C-S). The first cycle charge–discharge voltage profile in Figure 5a shows that the V_2_O_5_–C-L electrodes have a much lower polarization and therefore access an additional voltage plateau at 2.25 V on discharge. These electrodes achieve a specific capacity of 229.2 mA h g^–1^ at 20 mA g^–1^ in a voltage window of 2–4 V. This is in agreement with the energy storage mechanism of V_2_O_5_ cathodes detailed in Note S3. Due to polarization, the plateau at 2.25 V discussed above is pushed outside the cycling window for the two other electrodes, whose capacity was therefore capped at 131.6 mA h g^–1^ for the V_2_O_5_ electrode and 107.8 mA h g^–1^ for V_2_O_5_–C-S electrodes. The same evidence for two Li intercalation/ deintercalation redox reactions (in 3 cathodic peaks) is also captured using CV (Figure S12). The higher polarization and lower initial discharge capacities of the V_2_O_5_ electrode are probably due to poor electronic conductivity in the absence of carbon additives. In the case of V_2_O_5_–C-S, higher impedance could come from lower porosity decreasing ionic conductivity; however, over time, the polarization of these electrodes reduced, and as a result, after about 50 cycles, the full material capacity was accessed. This “activation” process has previously been reported by other groups using V_2_O_5_ electrodes.^32−37^

The activated binder-free V_2_O_5_–C-S electrode exhibits high cycling stability up to 500 cycles with 98.1% capacity retention in reference to the 101st fast cycles (when the electrode is considered “activated”). At the end of 500 cycles, the V_2_O_5_–C-S electrode (volumetric capacity of 190.4 mA h cm^–3^) significantly outperforms both the unaligned V_2_O_5_ electrode (11.7 mA h cm^–3^) and the aligned V_2_O_5_–C-L electrodes after 300 cycles (134.1 mA h cm^–3^). Note that the pure V_2_O_5_ electrode yields poor capacity retentions of 26.7% after 500 cycles (discharge capacity dropped from 43.8 to 11.7 mA h cm^–3^). The improved cycling stability of V_2_O_5_–C electrodes highlights the benefits of self-alignment in maintaining structural integrity, as shown by postmortem SEM images of cycled electrodes with negligible fiber alignment alteration (Figure S13).

For reference, classic nonaligned electrodes with V_2_O_5_–C were fabricated using 15% of Super P carbon black and 5% of binders (labeled as V_2_O_5_–C (15%C)). These electrodes have NF morphology similar to that of V_2_O_5_–C-S but achieved a higher specific capacity up to 139.4 mA h g^–1^ at 100 mA g^–1^. However, as discussed above, the volumetric performance of nanomaterial-based electrodes without using self-assembly tends to be low, with an initial volumetric capacity of 50.2 mA h cm^–3^ (the electrode morphology is shown in Figure S15).

Next, the influence of carbon decoration and self-assembly on the rate performance was studied. The discharge capacities for V_2_O_5_, V_2_O_5_–C-S, V_2_O_5_–C-L, and a reference electrode with 5% binder and 10% carbon black (V_2_O_5_–C (10%C)) are measured at current densities of 50(0.2C), 100(0.4C), 200(0.8C), 500(2C), and 1000(4C) mA g^–1^, after three formation cycles at 20 mA g^–1^. The short NF V_2_O_5_–C-S electrode stands out for its ultrahigh packing density, which translates into higher volumetric capacity as shown in Figure 5d, despite a similar gravimetric capacity to the V_2_O_5_ electrode (Figure 5c). At a current density of 50 mA g^–1^, these binder-free and carbon additive-free electrodes achieve a volumetric capacity of 192.3 mA h cm^–3^, which is higher than both the long V_2_O_5_–C NF electrode (175.6 mA h cm^–3^) and the pure V_2_O_5_ electrode (112.9 mA h cm^–3^). As the current density increases to 200 mA g^–1^, the V_2_O_5_–C-L electrode (141.7 mA h cm^–3^) retains capacity better than that of the V_2_O_5_–C-S electrode (9.9 mA h cm^–3^). This suggests that while the short fibers offer an initial packing density advantage, the longer fibers provide improved impedance, which is supported by Electrochemical Impedance Spectroscopy (EIS) data (Figure 5e), showing that the charge transfer resistance (*R*_ct_) is significantly lower in V_2_O_5_–C-L electrodes compared to V_2_O_5_–C-S electrodes and V_2_O_5_ electrodes (∼402, ∼10.5k, ∼14k Ω, respectively, fitting method detailed in Figure S19). In assessing the rate performance versus volumetric capacity, it is interesting to consider the reference sample of V_2_O_5_–C (10%C), which shares the morphology of short nanofibers as V_2_O_5_–C-S but contains an additional 10% conductive additive (Super P) and 5% CMC-SBR binder. The addition of carbon black enables the V_2_O_5_–C NFs to cycle reliably and stably at higher current densities, up to 4C (Figure 5c,d). However, this impedes nanofiber alignment (Figure S14), and even after extensive calendering, the V_2_O_5_–C (10%C) samples exhibit lower volumetric capacity performance compared to the binder-free and carbon additive-free V_2_O_5_–C-S samples at 0.2C. This highlights the opportunities in optimizing self-assembled binder-free electrodes for high volumetric energy density applications.

## Conclusions

In summary, this paper introduces a scalable self-assembly process to organize Li-ion battery cathode nanomaterials into densely packed electrodes. We demonstrate long-range self-alignment using aqueous suspension with both benchtop blade coater and pilot-scale continuous roll-to-roll slot-die casting. We coat electrodes up to 4 m in length and 11 cm in width and compare the self-assembly and electrochemical performance of nanorods with different lengths, aspect ratios, and carbon content. Using self-assembly, we increase the packing density of our optimized electrodes by 2-fold, resulting in significantly higher volumetric capacities (>190.4 mA h cm^–3^) even after 500 cycles with limited capacity fade (98.1%) despite using a binder and a conductive additive-free electrode. Our findings provide insights into how nanostructured materials can be applied in LIB electrodes to enhance both volumetric energy density and manufacturing scalability, promoting the development of more efficient and sustainable energy storage technologies.

## Experimental Section

### Synthesis of V_2_O_5_ Composite Nanofibers

The V_2_O_5_ nanofibers were synthesized using a hydrothermal process.^24,38,39^ First, 1.25 g of V_2_O_5_ powder (Sigma-Aldrich) was mixed with 86 mL of deionized water under stirring (∼400 rpm) at room temperature. Then, 17 mL of 30% H_2_O_2_ (Fisher Scientific) was added into the solution and stirred until a transparent orange color was obtained. Thereafter, 17 mL of 0.4 wt % GO water dispersion (Graphenea) was added slowly into the solution and continually stirred for another hour. The resultant solution was transferred into a ∼210 mL autoclave and maintained at 205 °C for 96 h. The as-obtained product was washed with deionized water by centrifugation and then freeze-dried overnight.

### Preparation of the Electrode

To prepare the binder-free V_2_O_5_ nanofiber composite electrode, the dried nanofibers were weighed and mixed with DI water at a concentration of 50 mg/mL and sonicated in a glass vial for complete dispersion until a viscous homogeneous solution was obtained. For comparison, the shorter nanofibers include an additional grinding step using a mortar and pestle before dispersion in water. The obtained solution was then blade-coated onto an aluminum/stainless-steel current collector. The sample with a binder and conductive additives was prepared by mixing the nanofibers, C45 Super P (Imerys (previously known as Timcal) graphite, carbon, and a water-based composite binder (carboxymethylcellulose, (CMC)) and styrene–butadiene rubber (SBR) (CMC/SBR = 1:1) in an 85:10:5 (as well as 80:15:5) weight ratio. Similarly, the mixture was dispersed in a small amount of DI water using an ultrasonicator to obtain a stable homogeneous ink before casting. All coated samples were then dried in the oven at 90 °C for at least an hour. The active material loading is ∼1 mg cm^–2^ for the binder-free electrodes and between 1 and 2 mg cm^–2^ for the electrodes with binders and Super P. The roll-to-roll coating was carried out on a Coatema Smartcoater 28.

### Characterizations of Nanofibers

#### X-ray Diffraction (XRD)

The powder patterns of the synthesized materials were determined by a Bruker D8 Advance powder XRD with CuKα radiation (*K*_α1_ = 1.540598, *K*_α2_ = 1.544426 nm). The 2θ range was set between 5° and 80°. The XRD spectrum of the V_2_O_5_–C sample was measured in 3000 steps of 2 s each, while the spectrum of the pure V_2_O_5_ sample was measured in 350 steps of 5 s each. The fiber powder sample was adhered to a glass slide using sticky glue and placed in the middle of the sample holder.

#### Scanning Electron Microscopy (SEM)

FEI Nova NanoSEM was used to study the nanofiber morphologies and the alignment pattern. The samples were mounted onto stubs using adhesive carbon tape and imaged at an accelerating voltage of 5 kV in secondary electron mode.

#### Zeta Potential

The measurements were run using a Malvern Zetasizer ZSP instrument with a disposable folded capillary cell. Nanowire dispersions were prepared at concentrations of 0.025 w/w% in DI water by ultrasonication. Each reported zeta potential distribution is an average of 3 separate measurements for the same sample, taking 12 runs per measurement.

#### Thermogravimetric Analysis (TGA)

The measurement was performed using a PerkinElmer Pyris 1 tool in an atmosphere of synthetic air with a flow rate of 20 mL/min. A solid sample mass of 1–3 mg was used. Nanoparticle samples were deposited from a slurry in isopropyl alcohol and dried on a hotplate at 80 °C before cooling and transferring for measurements. Samples were kept at room temperature for 15 min to stabilize before heating at a rate of 10 °C/min. A buoyancy correction was performed by running a blank sample under identical conditions.

### Battery Cycling

The dried electrodes were punched into 10 mm diameter circular electrode disks with an MTI electrode punch weighed and dried again in a vacuum oven at 120 °C for > 2 h before transferring into an Ar-filled glovebox (MBraun, H_2_O and O_2_ levels <0.5 ppm) for half-cell assembly. The standard LP57 electrolyte was used (1 M LiPF6, ethylene carbonate (EC)/ethyl methyl carbonate (EMC) 3:7 vol %)) with 0.5% vinylene carbonate (VC). The cell stack was crimped with an MSK-110 hydraulic crimping machine at 1000 psi. All assembled cells were rested for 24 h for electrolyte infiltration. Galvanostatic cycling studies were performed using a multichannel BioLogic BCS potentiostat/galvanostat. A current density of 250 mA g^–1^ was defined as the 1C rate and the remainder of the current density was applied according to this value. All cycles were performed with constant current charge and discharge within the voltage window of 2.0–4.0 V. Electrochemical impedance spectra (EIS) were recorded on a BioLogic multichannel VMP3 potentiostat with an amplitude of 5 mV and frequency ranging from 500 kHz to 1 mHz. The measured EIS data were fitted to the equivalent circuit models using the Python software package impedance.py.^40^

## Supporting Information Available

Data processing methods (alignment ratio), TGA, SEM, photos of roll-to-roll setup and coated films, CV, rate performance and long cycling performance, and EIS data (PDF)
