# Supplementary material for: Scalable Self-Assembly of Composite Nanofibers into High-Energy-Density Li-Ion Battery Electrodes
Source: ACS Nano. 2024 Sep 19;18(39):26799–806. doi: 10.1021/acsnano.4c07602 (PMC11447904; doi:10.1021/acsnano.4c07602)
Supplement: Supplementary file 1 — nn4c07602_si_001.pdf [file nn4c07602_si_001.pdf]

Supplementary Information

Scalable Self-assembly of Composite Nanofibres into High Energy Density Li-ion Battery Electrodes

Heng Wang[1]†, Yuling Xiong[2]†, Kate Sanders[1], Sul Ki Park[1], Jeremy J. Baumberg[2]* and Michael F.L. De Volder[1]*

1 Department of Engineering, University of Cambridge, Cambridge CB3 0FS, United Kingdom

2 Department of Physics, University of Cambridge, Cambridge CB3 0HE, United Kingdom

† These authors contributed equally to this work.

* Correspondence should be addressed to: jjb12@cam.ac.uk; mfld2@cam.ac.uk

Contents of Supplementary Information:

Note. S1: Thermogravimetric Analysis for carbon content estimation
Note. S2: Quantifying alignment through alignment ratio
Note. S3: Cyclic Voltammetry to identify specific reaction

Fig. S1: TGA of graphene oxide (commercially supplied) in air
Fig. S2: Examples of alignment ratio calculation from SEM images
Fig. S3: Effect of pH on NF alignment in drop-casted films
Fig. S4: Effect of solvent on NF alignment in drop-casted films
Fig. S5: Effect of concentration on drop-casted V_2O_5-C NF film
Fig. S6: Effect of concentration on drop-casted pure V_2O_5 NF film
Fig. S7: Blade coating V_2O_5 NF film
Fig. S8: Photos of the roll-to-roll slot die coating process
Fig. S9: Roll-to-roll slot die coating V_2O_5-C NF film
Fig. S10: Effect of grinding on blade coating V_2O_5-C films
Fig. S11: Variation in alignmemt ratios
Fig. S12: Cyclic Voltammetry of V_2O_5-C and V_2O_5-C
Fig. S13: Post-mortem Analysis
Fig. S14: Effect of binders & conductive additives on alignment of shorter V_2O_5-C NFs
Fig. S15: Effect of binders & conductive additives on alignment of longer V_2O_5-C NFs
Fig. S16: Effect of fibre alignment on the structural integrity of the films
Fig. S17: Long cycling data with gravimetric specific capacity and coulombic efficiency
Fig. S18: Rate performance test overview with gravimetric specific capacity
Fig. S19: Fitted EIS results in Nyquist plots with corresponding equivalent circuit models

Note. S1 | Thermogravimetric Analysis for carbon content estimation

Thermogravimetric Analysis (TGA) performed in the air (Fig 1e) shows that the V_2O_5-C nanowire samples synthesised with Graphene Oxide (GO) have a greater decrease in mass (to 97 ± 1 %), below 300°C compared to those where GO was not included in the synthesis (about 99%). However, the overall decrease in mass in the GO-derived carbon composite nanowire sample is still quite small (about 2 %). This aligned with our expectation since the post-synthesis washing procedure has likely removed a portion of the GO used in the synthesis. Comparing this with the TGA of the commercial GO starting material used in the synthesis (Fig S1), which shows three main mass loss events—one below 100°C (water loss), one between 200-300°C (loss of oxygen-containing functional groups), and one from around 500 to 700°C (pyrolysis of the remaining graphitic lattice)—the absence of the third decomposition peak confirms that the remaining carbon species in the nanofiber is integrated without forming a graphitic lattice, likely due to the low concentration of carbon in the V_2O_5-C NFs.

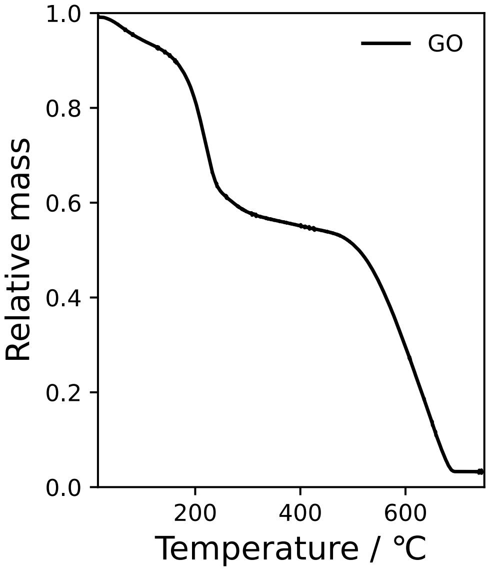

Fig. S1 | TGA of graphene oxide (commercially supplied) in air.

Note. S2 | Quantifying alignment through alignment ratio

SEM images showcasing aligned NFs (Fig S2a) exhibit an elliptical pattern in the FFT magnitude spectrum, while instances of randomly deposited NFs yield a spherical pattern. Subsequently, a spherical region is delineated on the FFT image (Fig S2b), and pixel intensities are cumulatively summed along radial lines. By plotting this radial summation against angles ranging from θ=0° to 180° (Fig S2c), the primary direction of alignment emerges distinctly. For NFs that are unidirectionally aligned, radial sum exhibits a sharp, singular peak, while in cases where multiple alignment domains coexist, or in instances of randomly oriented NFs, the radial sum broadens and becomes more flattened. The alignment can thus be quantified as follow[1,2]:

$$\text{Alignment ratio } \eta = \frac{\mathrm{I}_{max} - \mathrm{I}_{min}}{\mathrm{I}_{min}} \qquad Eqn. (1)$$

The absolute value of the alignment ratio does not reflect physical quantity and therefore is only used when compare between one another to indicate relative alignment. When comparing many images, the alignment ratio can be normalized by the maximum η value among the images, so that the most aligned sample is given a score of 1, and the least aligned sample is given a score of 0.

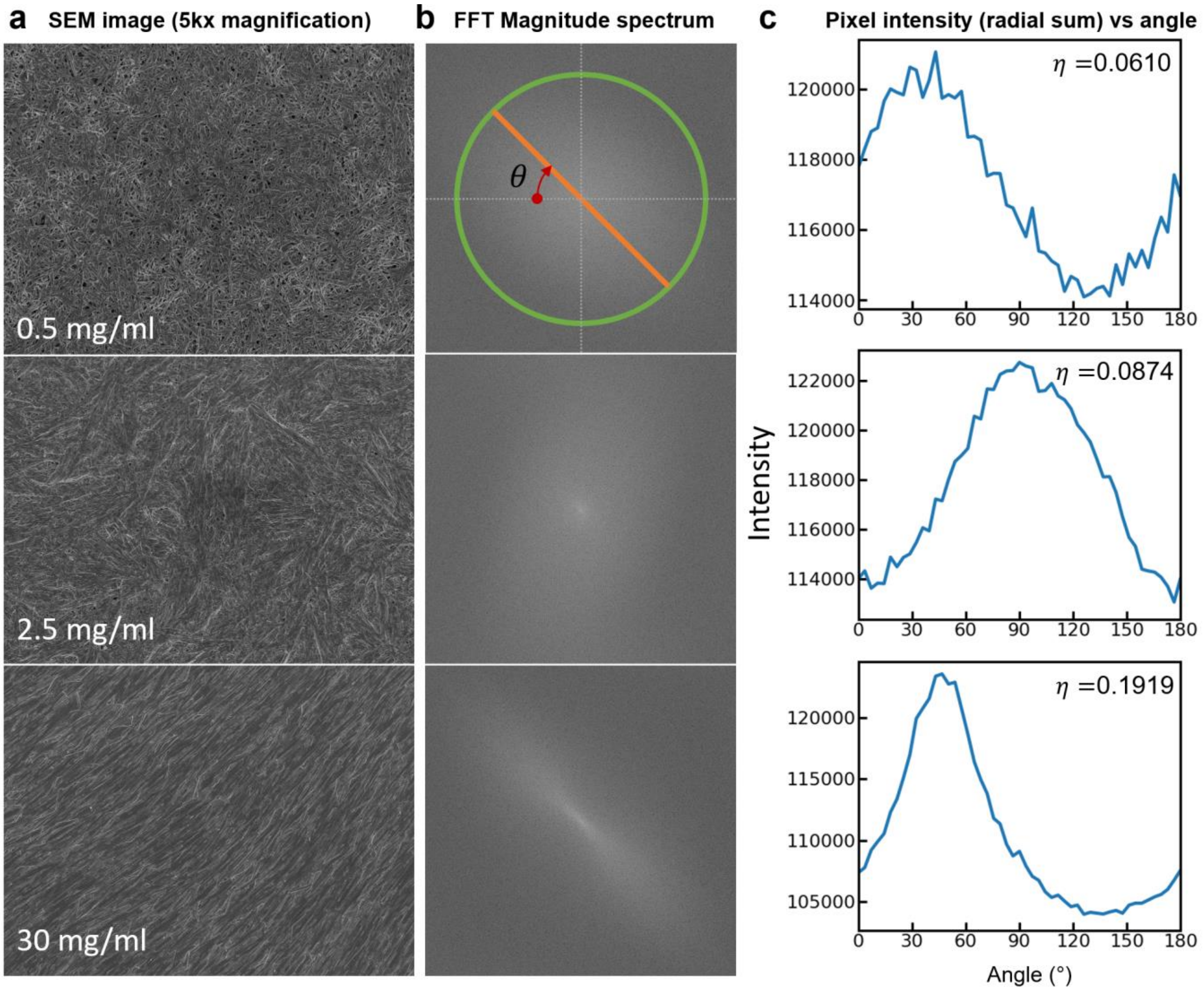

Fig. S2 | Examples of alignment ratio calculation from SEM images: (a) Input SEM image of drop-casted V_2O_5-C NFs films at 5000×magnification. (b) FFT magnitude spectrum obtained from input SEM image. (c) Sum pixel intensity versus angle and calculated alignment ratio η.

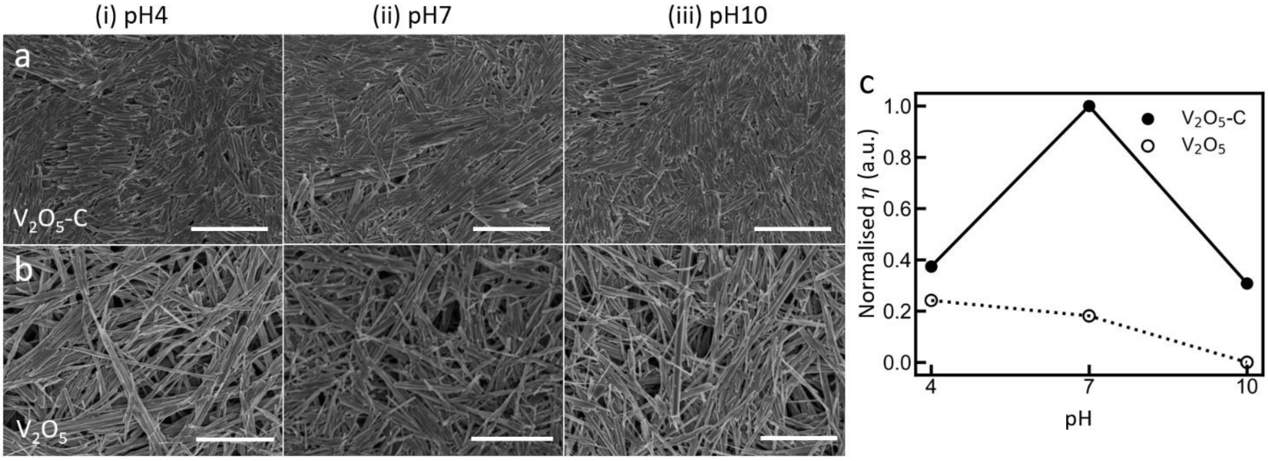

Fig. S3 | Effect of pH on NF alignment in drop-casted films: (a) SEM images showing V_2O_5-C NFs and (b) pure V_2O_5 NFs alignment at concentration of 5 mg/ml under different pH. Scale bar: 2 μm. (c) Alignment ratio η of V_2O_5-C and pure V_2O_5 NFs film against pH. η value averaged across three different positions on each sample, and normalised by the maximum value of six samples.

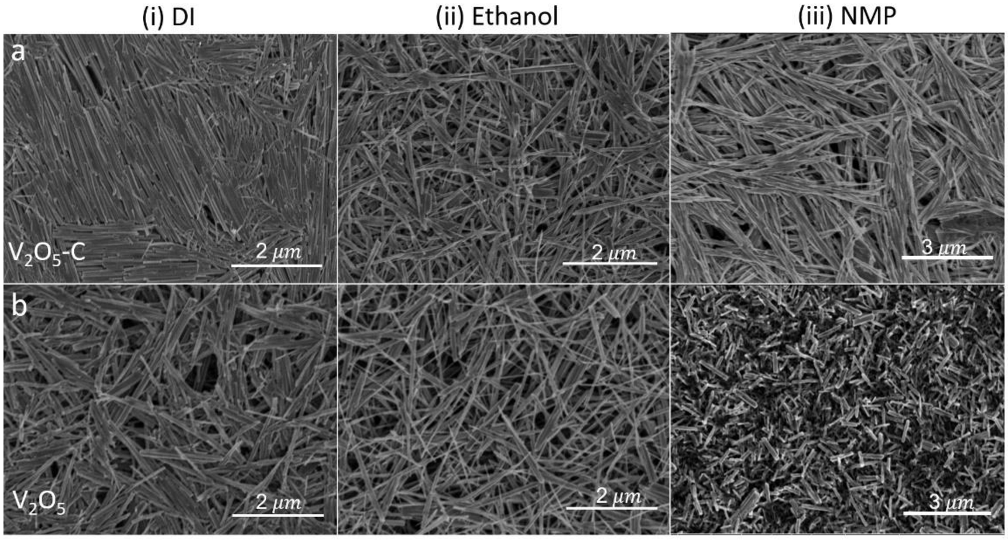

Fig. S4 | Effect of solvent on NF alignment in drop-casted films: (a) SEM images showing V_2O_5-C NFs and (b) pure V_2O_5 NFs alignment in different solvents. Concentrations: 5 mg/ml.

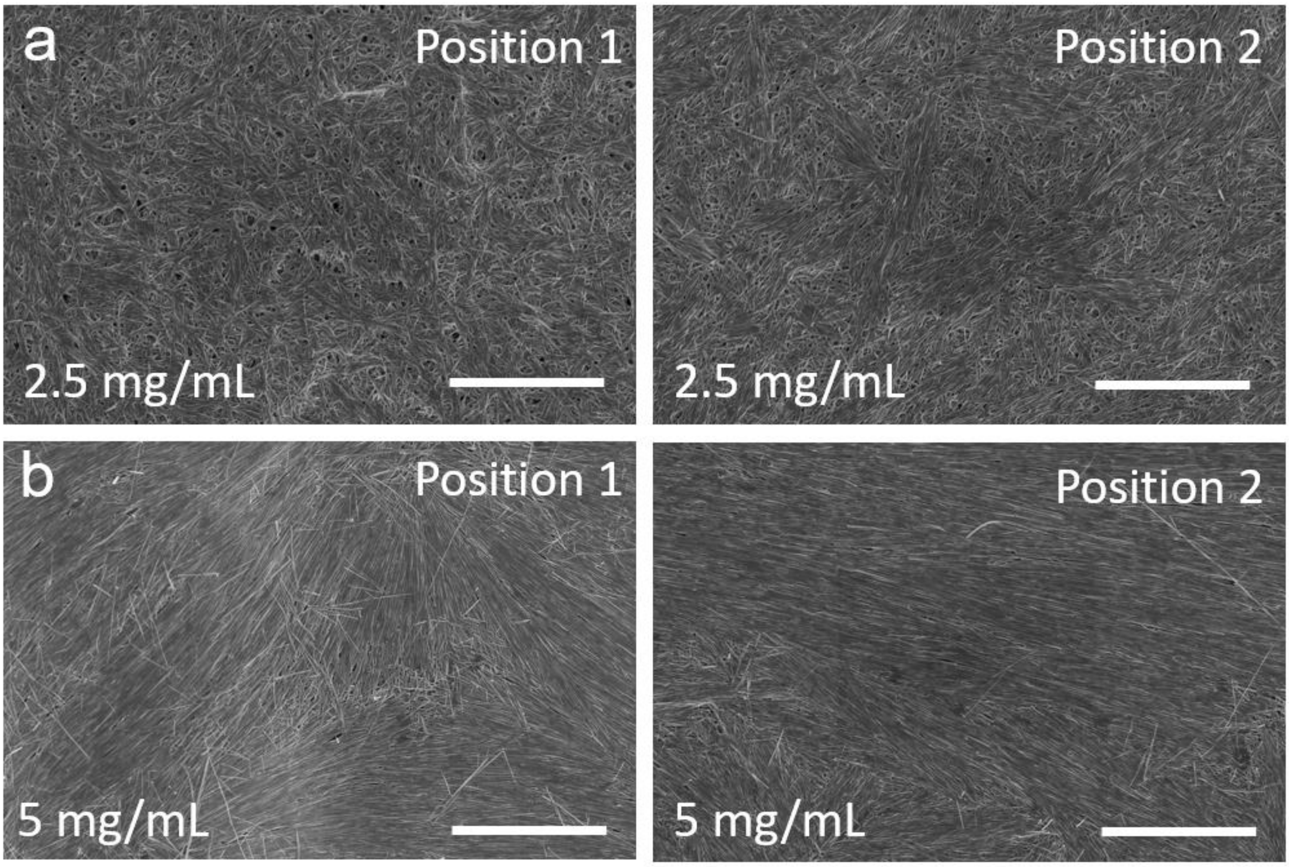

Fig. S5 | Effect of concentration on drop-casted V_2O_5-C NF film: SEM images showing V_2O_5 NF alignment at a concentration of (a) 2.5 mg/mL and (b) 5 mg/mL in water. Scale bar: 5 μm.

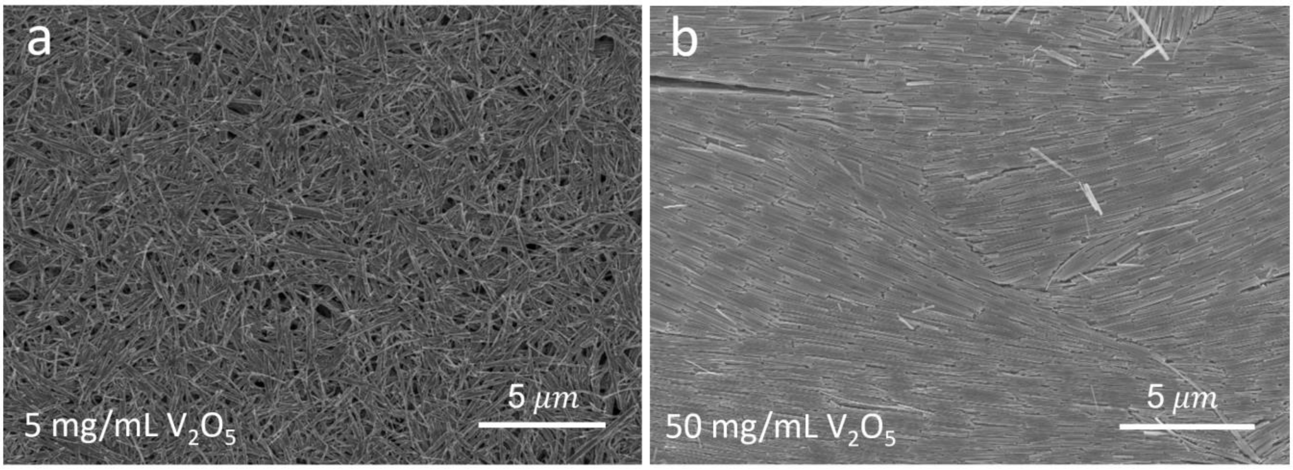

Fig. S6 | Effect of concentration on drop-casted V_2O_5 NF film: SEM images showing V_2O_5 NF alignment at a concentration of (a) 5 mg/mL and (b) 50 mg/mL in water. Scale bar: 5 μm.

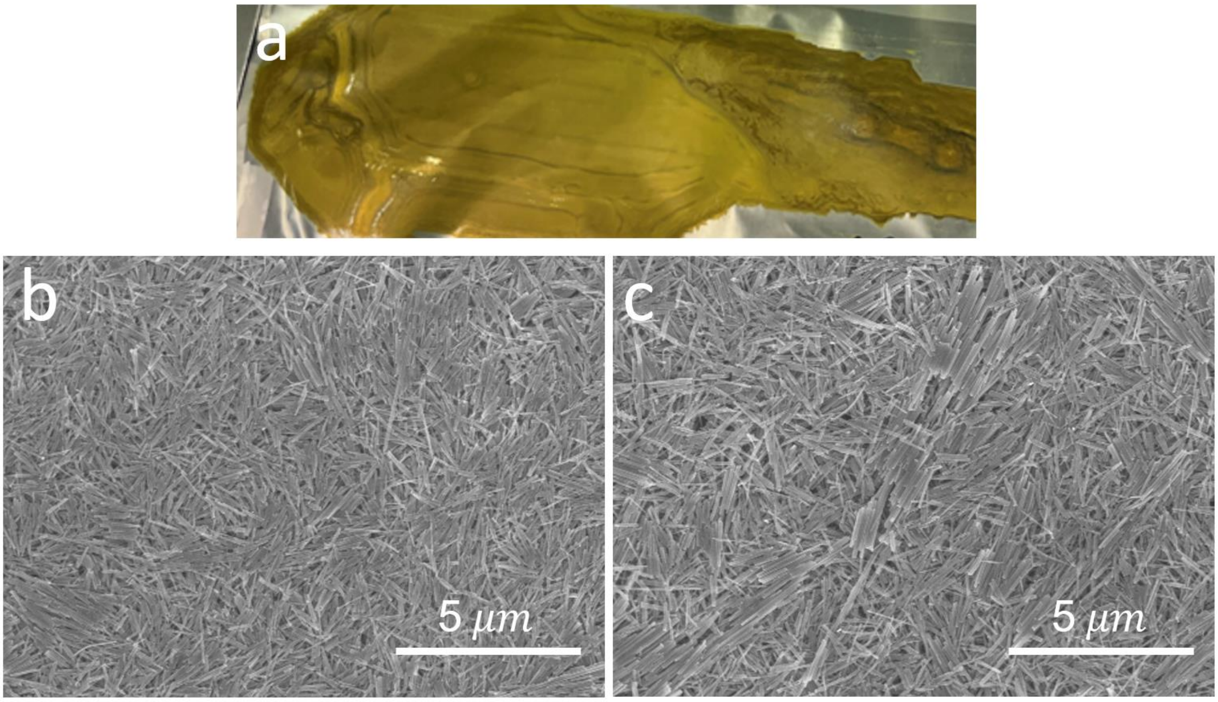

Fig. S7 | Blade coating V_2O_5 NF film: (a) Photo of the blade coating V_2O_5 NF film prepared using 50 mg/mL NF water dispersion. (b-c) SEM images taken at two different position on sample in (a) show random orientation of V_2O_5 NFs.

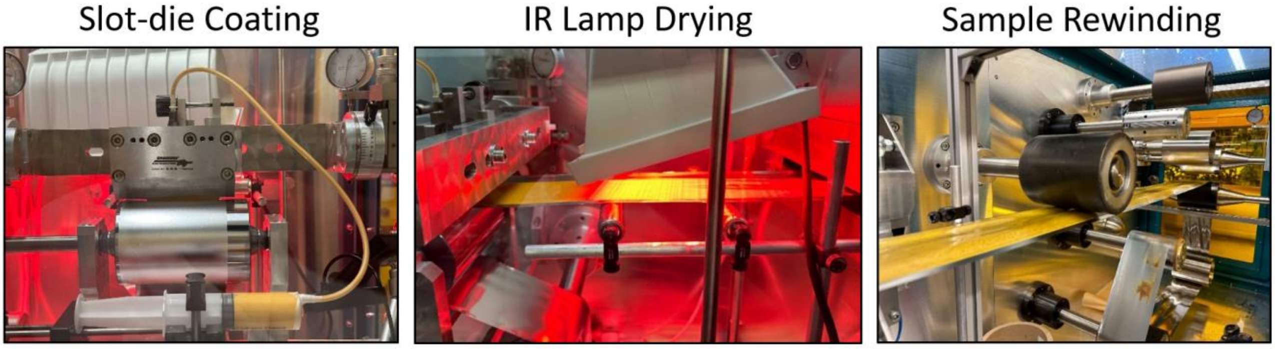

Fig. S8 | Photos of the roll-to-roll slot die coating process: producing V_2O_5-C film on Al substrate prepared using 25 mg/mL NF water dispersion

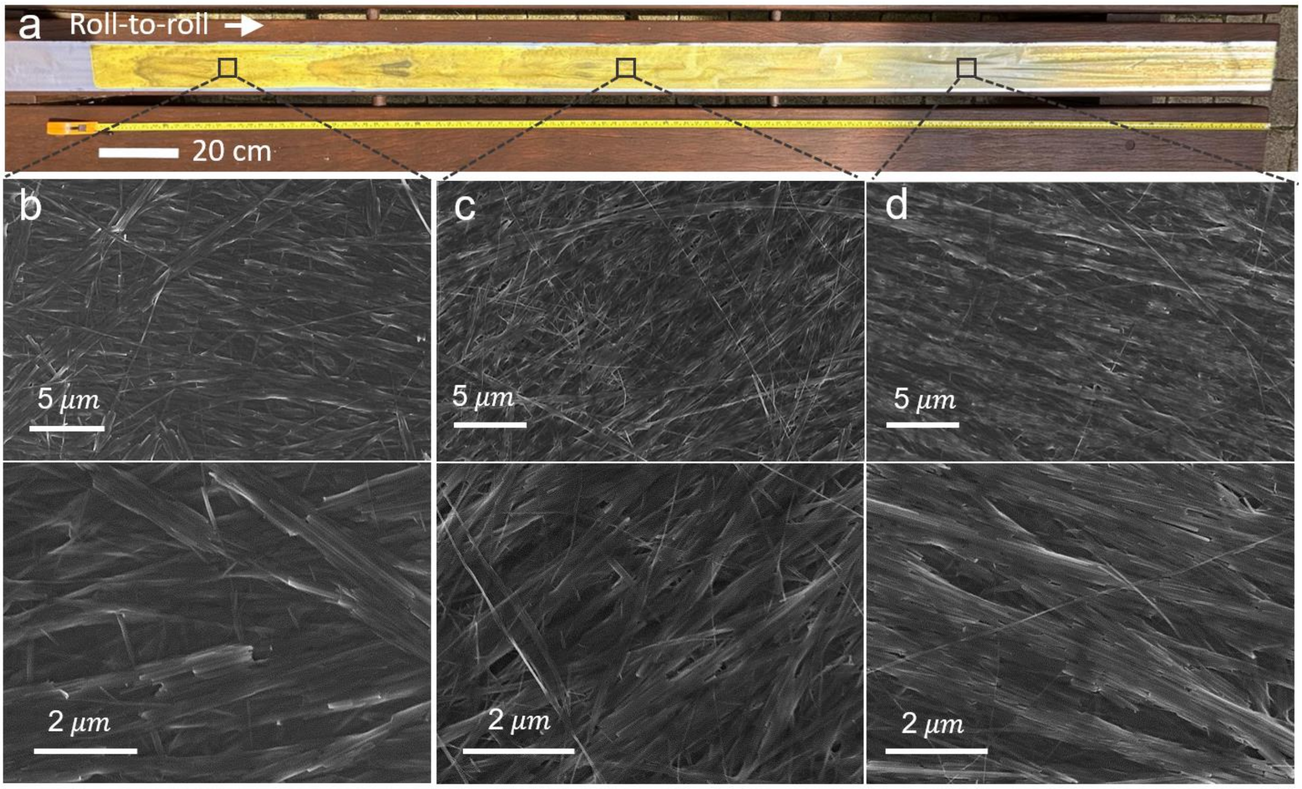

Fig. S9 | Roll-to-roll slot die coating V_2O_5-C NF film: (a) Photo of the slot-die coating V_2O_5-C film on Al substrate prepared using 25 mg/mL NF water dispersion. (b-d) SEM images taken at three different position on sample in (a) showing alignment of V_2O_5-C NFs. Top: SEM images taken at 2.5 k× magnification. Bottom: zoom-in SEM images taken at 10 k× magnification.

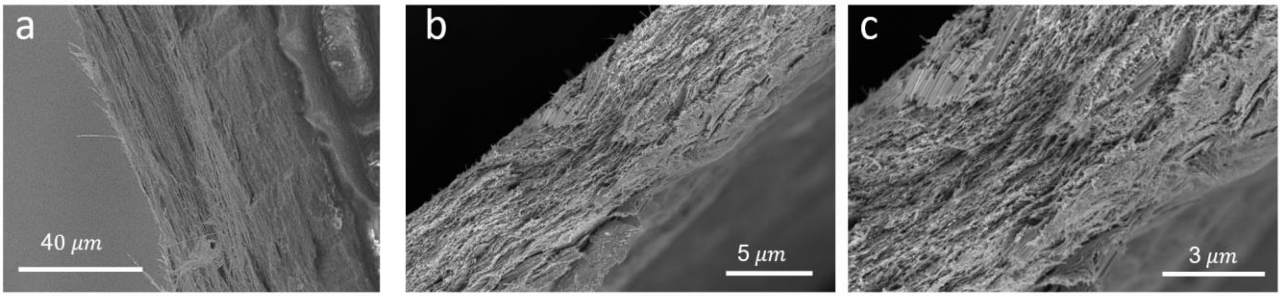

Fig. S10 | Effect of grinding on blade coating V_2O_5-C films: (a) Cross-sectional SEM image of films made of long NFs and (b) short NFs (l<5 μm) prepared using 50 mg/mL NF disperssion. (c) SEM of blace coating film made of short NFs at high magnification.

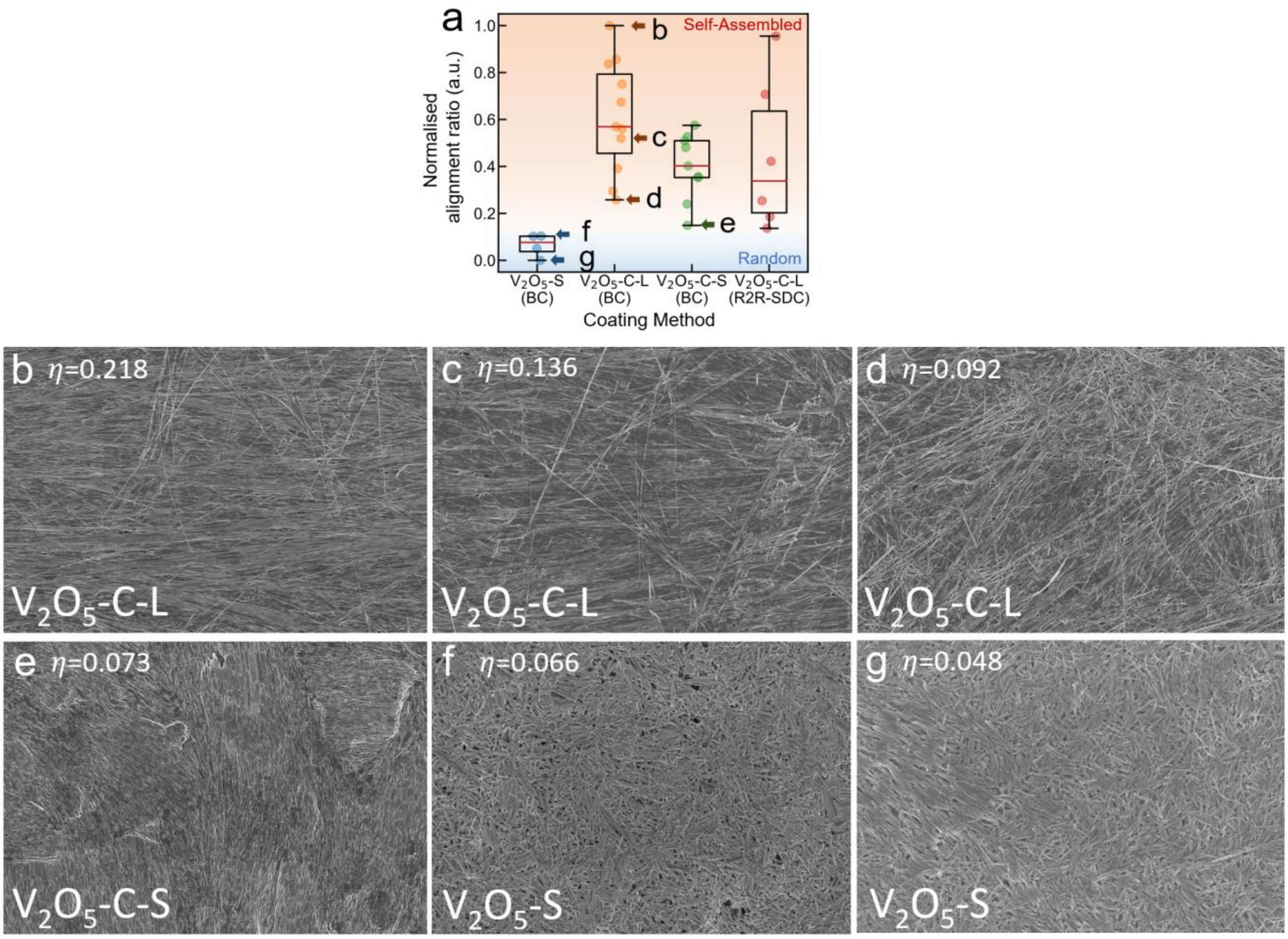

Fig. S11 | Variation in alignmemt ratios: (a) Normalized alignment ratio comparisons for various coating methods. Arrows highlight data with corresponding SEM images at 5000×magnification shown in panels (d-g). (b-d) SEM images from three sites on a blade-coated film of long V_2O_5-C NFs, demonstrating a reduction in alignment ratio due to the increased presence of detached nanofibers. (e) SEM image from a blade-coated film of short V_2O_5-C NFs, depicting a low alignment ratio influenced by multi-domain nanofibers with varied orientations. (f-g) SEM images from two sites on a blade-coated film of short V_2O_5 NFs, showing low alignment ratios due to randomly oriented nanofibers.

Note. S3 | Cyclic Voltammetry to Identify Specific Reaction

As the Lithium intercalates into the structure, 3 cathodic peaks are visible at around 3.4 V, 3.2 V and 2.3 V in the V_2O_5-C NF electrode, each corresponding to Eq. (1-3) and voltage plateau during the discharge. For V_2O_5 NF electrode, the 3rd cathodic peak is less obvious due to the poor electrical conductivity. The high overpotential shifts the cathodic peaks (as voltage plateau) to lower voltage, causing the structure to be less capable of accessing the secondary Li ion intercalation for additional discharge capacity.

$$V_2O_5 + 0.5Li^+ + 0.5e^- = \varepsilon - Li_{0.5}V_2O_5 \quad (1)$$

$$Li_{0.5}V_2O_5 + 0.5Li^+ + 0.5e^- = \delta - Li_{1.0}V_2O_5 \quad (2)$$

$$Li_{1.0}V_2O_5 + 1.0Li^+ + 1.0e^- = \gamma - Li_{2.0}V_2O_5 \quad (3)$$

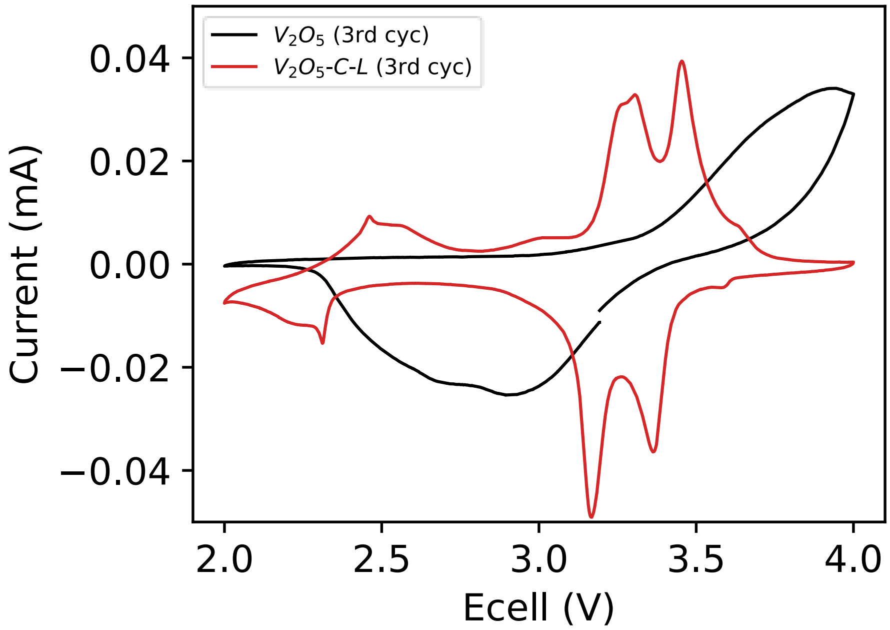

Fig. S12 | Cyclic Voltammetry of V_2O_5 and V_2O_5-C.

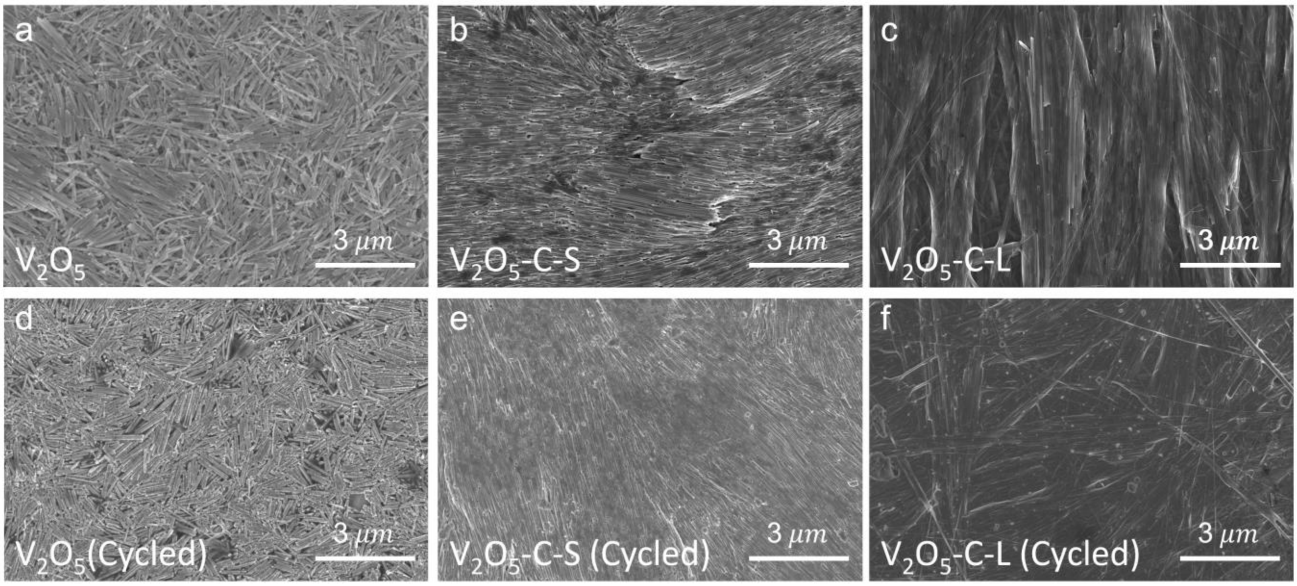

Fig. S13 | Post-mortem Analysis: (a-c) SEM images showing the alignment of the blade-coated NF electrodes before cycling for reference. SEM images are taken from dissembled cells of (d) V_2O_5 NF electrode after 500 cycles, (e) V_2O_5-C-S electrode after 200 cycles, (f) V_2O_5-C-L electrode after 500 cycles.

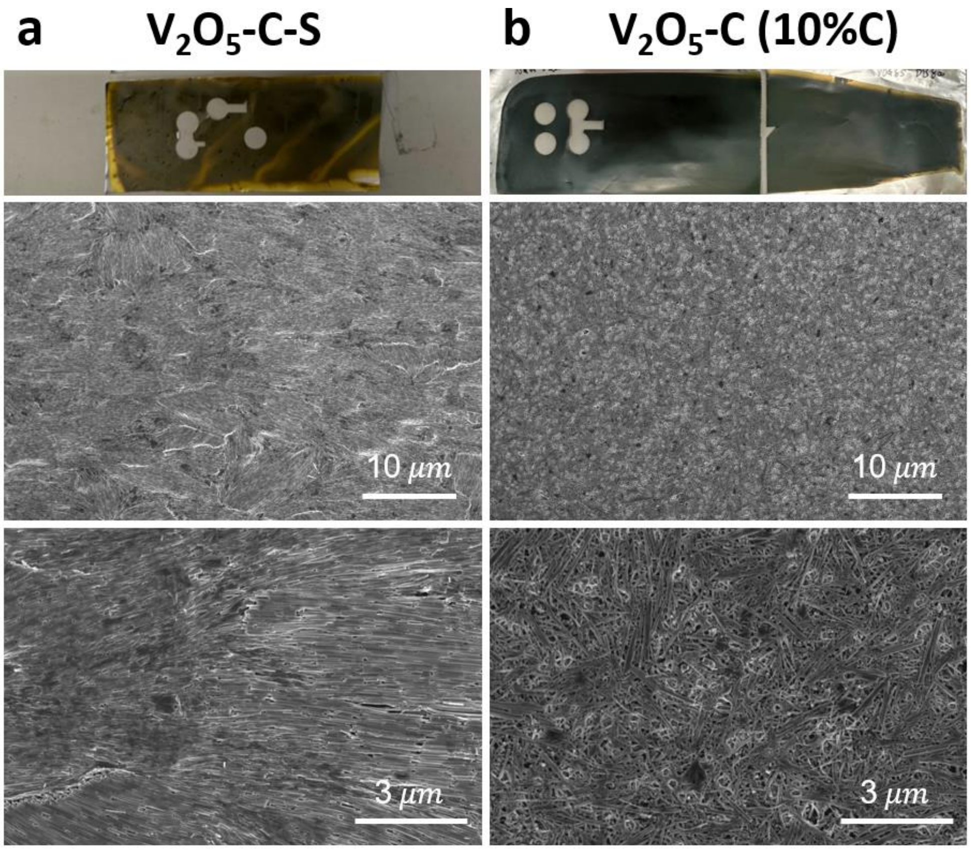

Fig. S14 | Effect of Binders & Conductive Additives on Alignment of Shorter V_2O_5-C NFs (l<5 μm): Photo and SEM images on the calendered films of (a) V_2O_5-C-S NFs (b) V_2O_5-C with 5% binder and 10% carbon black

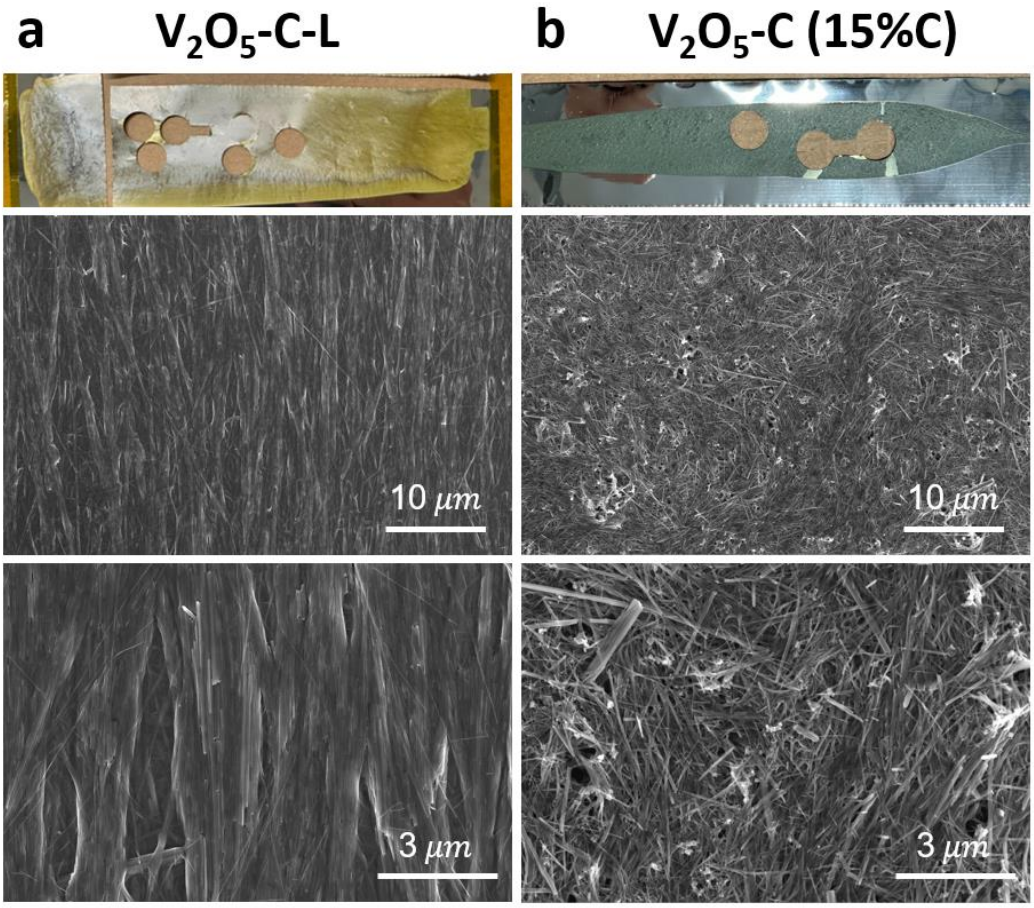

Fig. S15 | Effect of Binders & Conductive Additives on Alignment of Longer V_2O_5-C NFs (l>5 μm): Photo and SEM images on the (a) calendered film of V_2O_5-C-S NFs (b) uncalandered (due to brittleness) film of V_2O_5-C with 5% binder and 15% carbon black

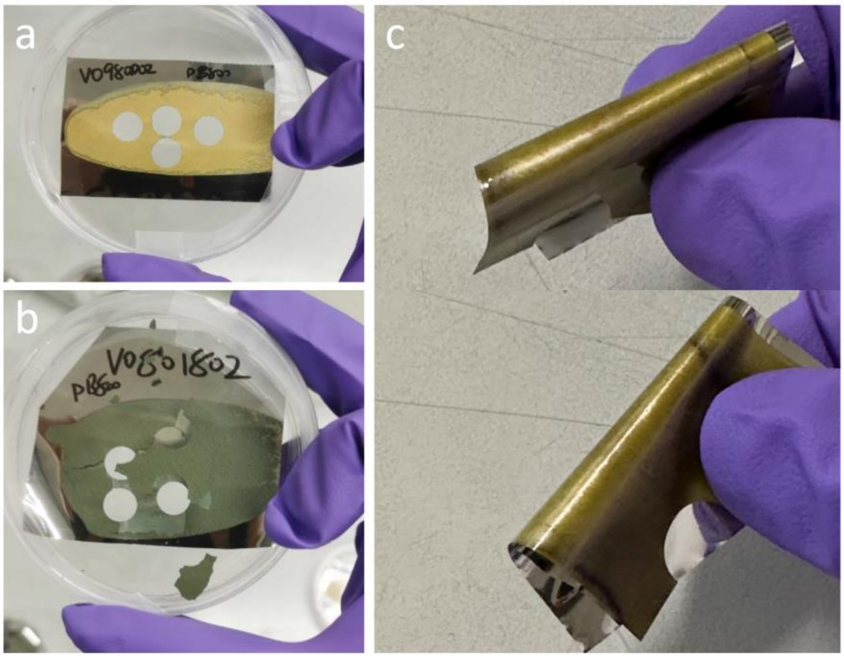

Fig. S16 | Effect of Fiber Alignment on the Structural Integrity of the Films: (a-b) Brittle films of V_2O_5 NF electrode films even with addtion of up to 3% binders (c) Structrually robust binder-free V_2O_5-C NF film as close-packed NFs are held together to preserve their structual integrity.

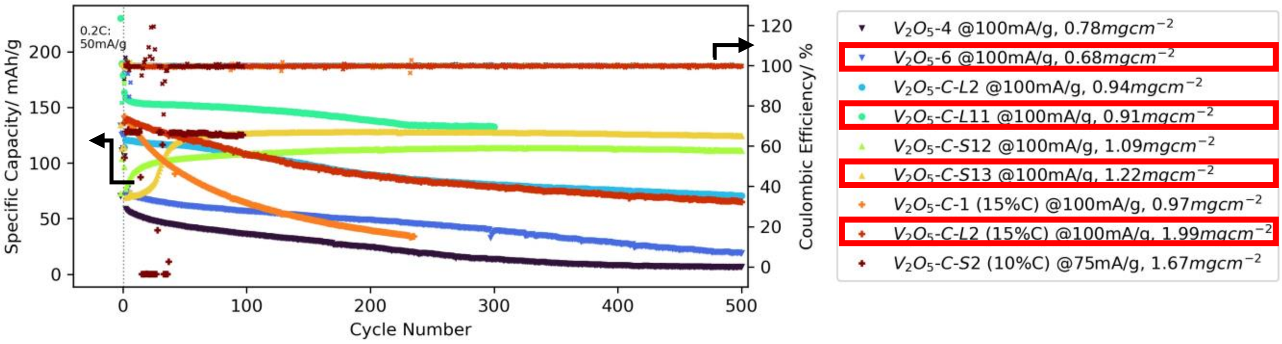

Fig. S17 | Long Cycling Data with Gravimetric Specific Capacity and Coulombic Efficiency: Samples highlighted in red box presented in **Fig. 5b** of the manuscript; V_2O_5 = pure V_2O_5 NF electrode; V_2O_5-C-L = longer (length >5 µm) carbon-decorated V_2O_5 NF electrode; V_2O_5-C-S = shorter (length <5 µm) carbon-decorated V_2O_5 NF electrode; (X% C) = electrode with X wt% of Super P conductive additive and 5% of CMC-SBR binder

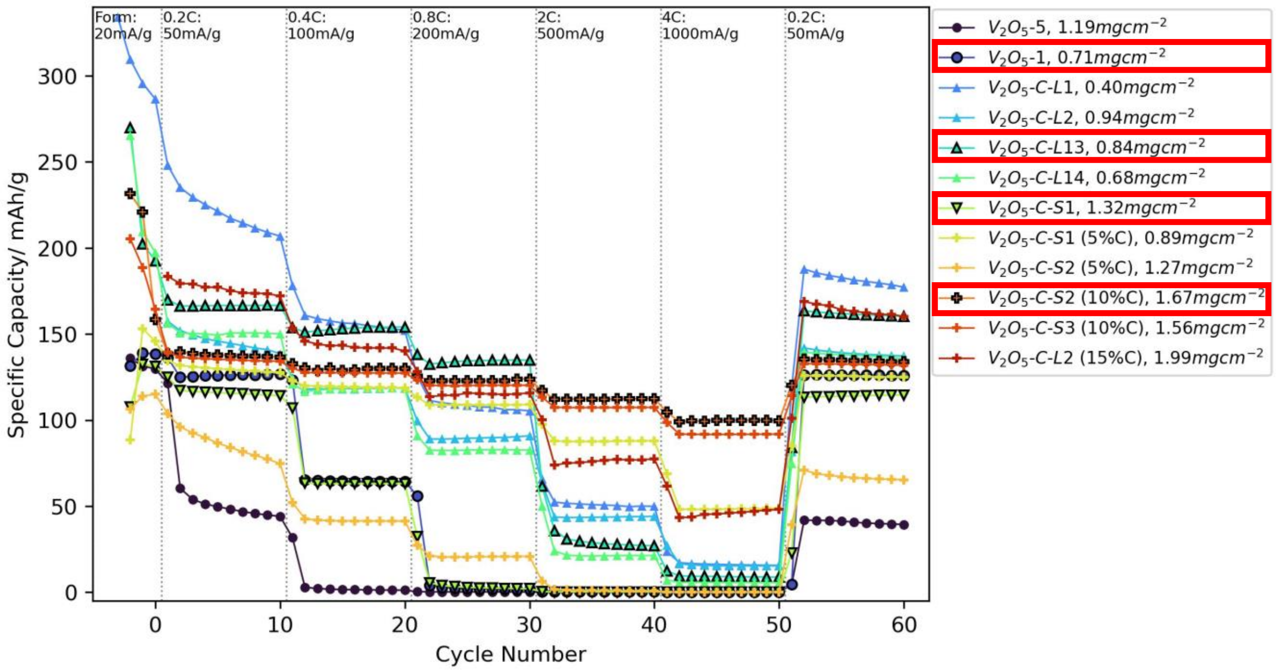

Fig. S18 | Rate Performance Test Overview with Gravimetric Specific Capacity: Samples highlighted in red box presented in **Fig. 5c&d** of the manuscript; V_2O_5 = pure V_2O_5 NF electrode; V_2O_5-C-L = longer (length >5 µm) carbon-decorated V_2O_5 NF electrode; V_2O_5-C-S = shorter (length <5 µm) carbon-decorated V_2O_5 NF electrode; (X% C) = electrode with X wt% of Super P conductive additive and 5% of CMC-SBR binder

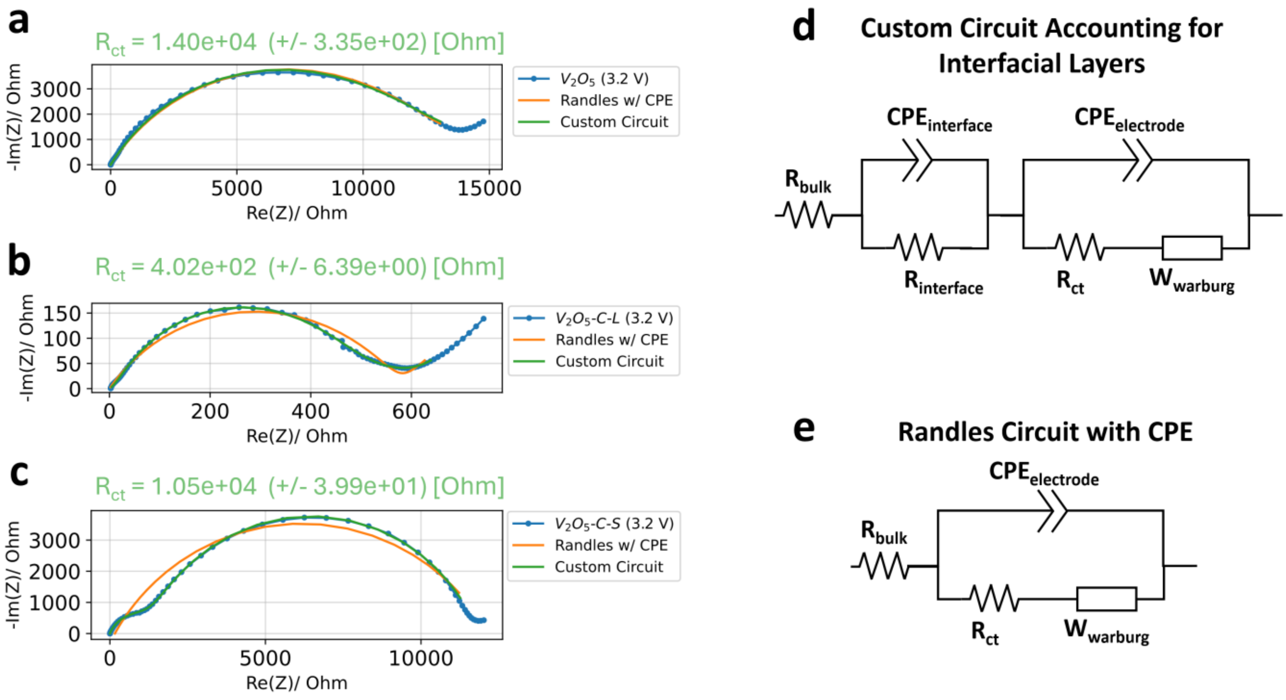

Fig. S19 | Fitted EIS Results in Nyquist Plots with Corresponding Equivalent Circuit Models: Fitted Electrochemical Impedance Spectroscopy results of (a) V_2O_5, (b) V_2O_5-C-L, and (c) V_2O_5-C-S at 3.2V after formation cycles. The R_{ct} values are calculated by fitting (d) a custom equivalent circuit model [3] which include additional resistance and CPE (Constant Phase Element) on top of (e) the Randles Circuit with CPE to account for the interfacial layer.

Reference

1. Modarres, M. H., Kosasih, F. U., Ducati, C. & De Volder, M. Self-Assembly of rGO Coated Nanorods into Aligned Thick Films. *Adv. Mater. Interfaces* **6**, 1–7 (2019).
2. Ayres, C. *et al.* Modulation of anisotropy in electrospun tissue-engineering scaffolds: Analysis of fiber alignment by the fast Fourier transform. *Biomaterials* **27**, 5524–5534 (2006).
3. Choi, W., Shin, H.-C., Kim, J. M., Choi, J.-Y. & Yoon, W.-S. Modeling and Applications of Electrochemical Impedance Spectroscopy (EIS) for Lithium-ion Batteries. *J. Electrochem. Sci. Technol* **11**, 1–13 (2020).
